# Advanced Biventricular Heart Failure due to Left Ventricular Noncompaction Cardiomyopathy Leading to the Formation of a Gastric Bezoar: The Implications of Heart Failure on the Gastrointestinal Tract

**DOI:** 10.1155/2018/4386025

**Published:** 2018-10-01

**Authors:** Daria Yunina, Dikshya Sharma, Richard Fazio, Hossam Amin, Yuriy Tsirlin, Vijay Shetty

**Affiliations:** ^1^Department of Medicine, Maimonides Medical Center, Brooklyn, NY, USA; ^2^Division of Gastroenterology, Department of Medicine, Maimonides Medical Center, Brooklyn, NY, USA; ^3^Department of Cardiology, Department of Medicine, Maimonides Medical Center, Brooklyn, NY, USA; ^4^Department of Medicine, SUNY Downstate School of Medicine, Brooklyn, NY, USA

## Abstract

Congestive heart failure (CHF) is a chronic disease process affecting multiple organ systems and is associated with significant morbidity and mortality. We report a case of a 43-year-old male with a history of unspecified cardiomyopathy who presented to the hospital with abdominal pain, distention, and nausea for 4 months. He was diagnosed with left ventricular noncompaction and gastroparesis. While symptoms of dyspnea, orthopnea, or increasing peripheral edema are the first that come to mind when thinking of a CHF exacerbation, we must broaden our scope to include such things as nausea, vomiting, abdominal pain, and bloating which can also indicate worsening cardiac function. This case report highlights the significant yet often forgotten gastrointestinal (GI) symptoms that result from advanced biventricular heart failure, with emphasis on impaired gastric and intestinal motility.

## 1. Introduction

Congestive heart failure (CHF) is a chronic disease process affecting multiple organ systems and is associated with significant morbidity and mortality. In the United States (U.S.) alone, its prevalence is close to 6 million, with a yearly incidence of over 550,000 and a death rate of 300,000 per year [1]. This case report will highlight the significant yet often forgotten gastrointestinal (GI) symptoms that result from advanced biventricular heart failure, with emphasis on impaired gastric and intestinal motility.

## 2. Case Presentation

A 43-year-old male with past medical history of hypertension, depression, and an unspecified cardiomyopathy presented to the hospital with complaints of abdominal pain, distention, and nausea for 4 months, worse in recent weeks. The patient had multiple episodes of vomiting with inability to tolerate anything by mouth. His primary care doctor prescribed metoclopramide and famotidine, which provided minimal relief. In parallel to his GI symptoms, the patient also described symptoms of fatigue and worsening shortness of breath on exertion. He denied chest pain, palpitations, diarrhea, constipation, or increased swelling of the legs. The patient was a former cigarette smoker and drank alcohol socially.

On physical examination, there was jugular venous distention and trace peripheral edema. Auscultation of the lungs was significant for mildly diminished breath sounds in bilateral lower lobes with bibasilar crepitations. Cardiac auscultation was significant for a 2/6 holosystolic murmur of mitral and tricuspid regurgitation. Bowel sounds were normal. The abdomen was soft but mildly distended with diffuse tenderness. There was no guarding or rigidity.

Initial laboratory findings showed a B-type natriuretic peptide (BNP) of 1374 pg/ml consistent with volume overload. The basic metabolic panel was normal. Venous blood gas showed an elevated lactic acid level of 3.2 mmol/l suggesting tissue hypoperfusion. Complete blood count (CBC) and coagulation panel showed a platelet count of 66 K/Ul and an international normalized ratio (INR) of 2.3 (the patient was not taking anticoagulants prior to admission). These findings indicated some degree of liver dysfunction which was confirmed by abnormal liver function tests (LFTs) that showed an albumin of 3 g/dl, direct bilirubin of 1.9 mg/dl, total bilirubin of 4.0 mg/dl, aspartate aminotransferase of 98 IU/l, alanine aminotransferase of 121 IU/l, and an alkaline phosphatase of 87 IU/l. Chest radiograph showed cardiac enlargement. The patient was admitted for further workup of abdominal pain and management of acute decompensated heart failure.

**Figure 1:**
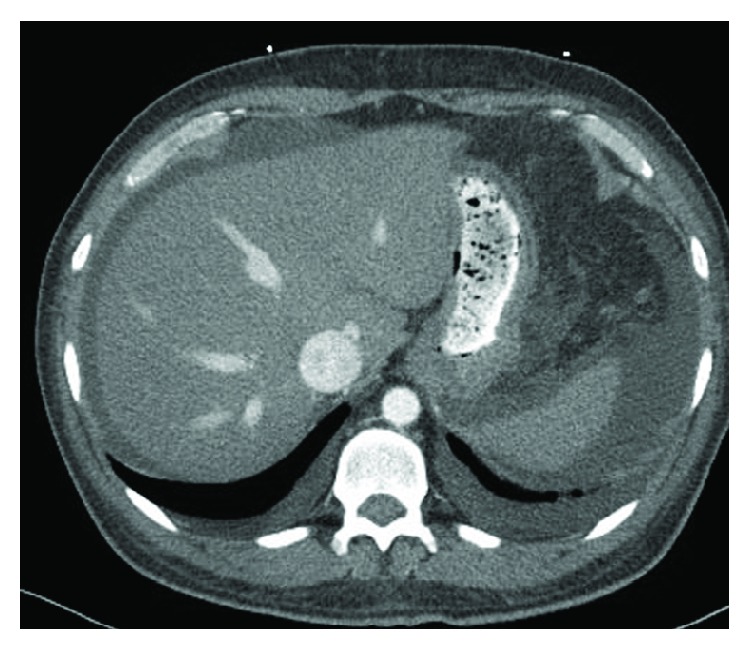
CT scan of the abdomen.

Computerized tomography (CT) of the abdomen and pelvis with oral and intravenous (IV) contrast was performed showing wall thickening of the cardia of the stomach, a moderate amount of abdominal ascites, and diffuse anasarca and thickening within the proximal sigmoid colon (Figure 1). Abdominal ultrasound (US) showed fatty infiltration of the liver and distention of the intrahepatic inferior vena cava (IVC) and hepatic veins. Echocardiogram (ECHO) showed a severely decreased left ventricular (LV) systolic function with ejection fraction (EF) of 21–25%. There was global cardiomyopathy, moderate LV diastolic dysfunction, and severe mitral and tricuspid valve regurgitation. The right ventricle (RV) was moderately dilated and hypokinetic. The patient's last known EF was reported to be 40% a year ago.

Cardiology evaluated the patient and recommended medical optimization and diuresis with plans for eventual right and left heart catheterization once he was euvolemic. Gastroenterology evaluated the patient for his abdominal pain, nausea, vomiting, and elevated LFTs. The patient's liver function was suggestive of cirrhosis. Viral hepatitis panel, iron studies, serum ceruloplasmin, alpha-1-antitrypsin, anti-mitochondrial antibodies, anti-smooth muscle antibodies, and anti-nuclear antibodies were unremarkable, ruling out infectious and autoimmune causes, making the etiology of his cirrhosis and abdominal complaints likely cardiogenic in origin.

**Figure 2:**
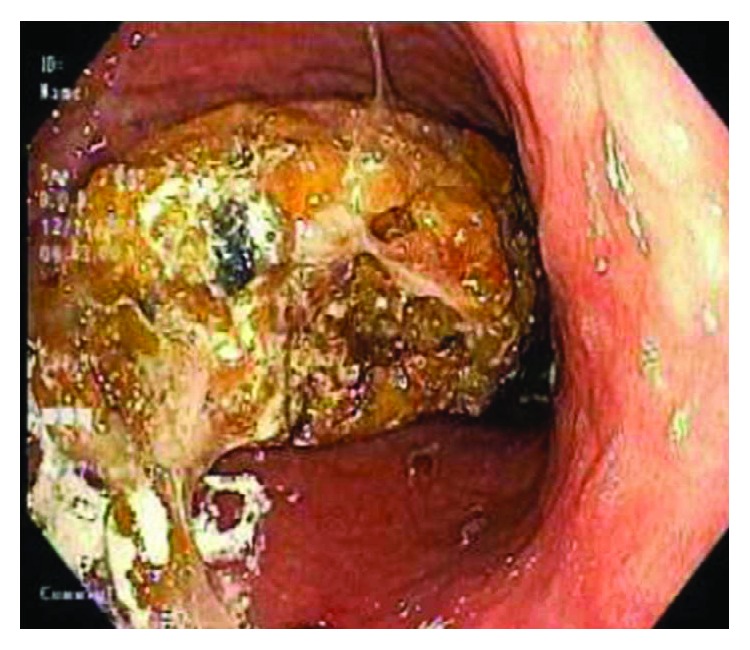
Gastric bezoar.

Upper endoscopy was performed on hospital day 2 to rule out peptic ulcer disease as a cause of his nausea and vomiting, revealing desquamation of the esophageal mucosa in linear streaks and a large gastric bezoar obstructing the view of the underlying mucosa (Figure 2).

**Figure 3:**
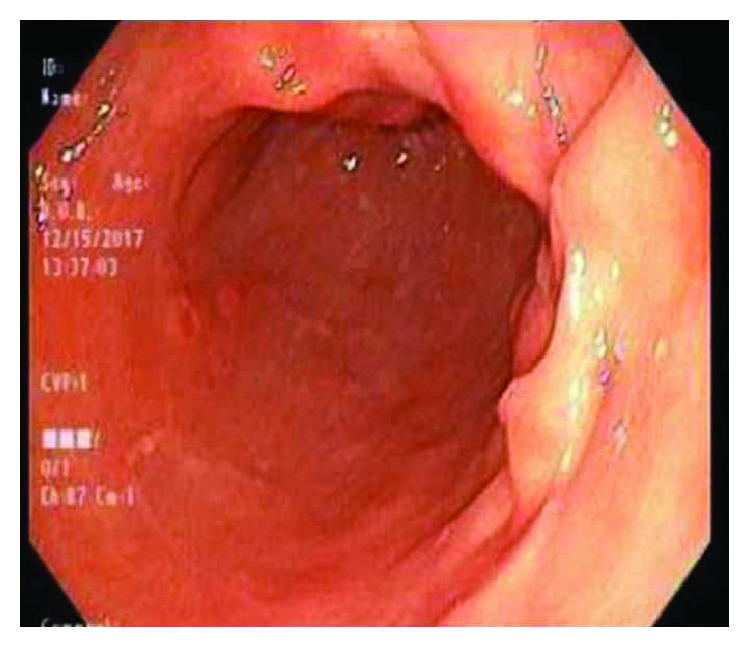
Body of the stomach.

After the endoscopy, the patient was given one can of Pepsi Cola and otherwise kept nil per os (NPO) for repeat endoscopy the following day with plans of possible bezoar removal. Repeat esophagogastroduodenoscopy (EGD) showed no evidence of the bezoar (Figure 3).

Despite resolution of the bezoar, the patient continued to have complaints of intermittent nausea. Right and left heart catheterization was performed on hospital day 8, showing normal coronaries, reduced cardiac output, and mild pulmonary hypertension. It was suspected that the reason pulmonary pressures were noted to be lower than expected was because the patient had received aggressive dieresis in the days leading up to the catheterization and was relatively euvolemic.

**Figure 4:**
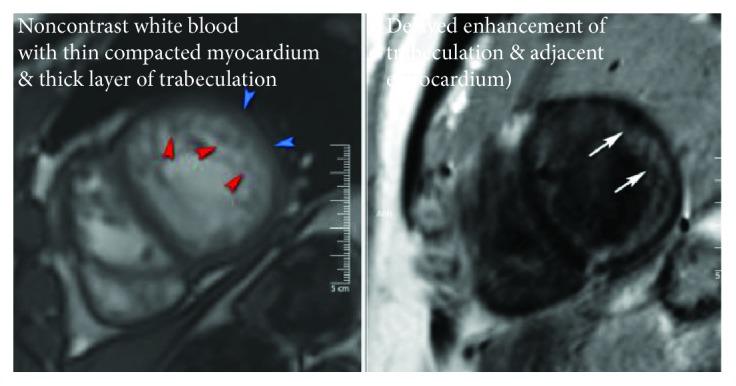
CMR showing compacted myocardium and trabeculated layer.

His medications were optimized and he was fitted for a LifeVest prior to discharge from the hospital. After discharge, he underwent cardiac magnetic resonance imaging (CMR) which revealed a diagnosis of left ventricular noncompaction cardiomyopathy (LVNC) (Figure 4). Cardiac output of the LV in cardiac MRI was measured to be 4.08 l/min with and EF of 28%. The RV was moderately dilated and noted to have an EF of 27%.

He also underwent a nuclear medicine gastric emptying study, which showed 24% gastric emptying at 90 minutes with the majority of the radioactive meal remaining in the proximal stomach. This study confirmed gastroparesis in our patient, presumably secondary to worsening cardiac function in the presence of biventricular failure due to noncompaction cardiomyopathy.

## 3. Discussion

As the prevalence of heart failure increases, so does the number of organ systems we see being affected by the disease. While symptoms of dyspnea, orthopnea, or increasing peripheral edema are the first that come to mind when thinking of a CHF exacerbation, we must broaden our scope to include such things as nausea, vomiting, abdominal pain, and bloating which can also indicate worsening cardiac function. Here, we look into the pathophysiology of the failing heart and its effect on the structure and function of the gastrointestinal system.

In the stomach, there can be notable thickening of the mucosa and areas of vascular ectasias [2]. The small intestine and the colon may display sonographic evidence of bowel wall edema with increased intestinal wall thickness. Increased distance between the basal membrane of the intestinal cells and the capillary blood flow leads to impaired nutrition of the enterocytes resulting in decreased absorptive function of the bowel wall [2]. The sigmoid colon is of particular importance as increased thickness positively correlates with an increase in blood concentrations of C-reactive protein and leukocytes [2].

Naturally, changes in the structure of the GI tract affect its function. The intestine is one of the most perfused organs at rest, receiving approximately 25% of the cardiac output [2]. At maximal exercise intensity, gut perfusion drops to 4% of the cardiac output [3]. The sympathetic nervous system innervates the splanchnic circulation, thus at a time of increased sympathetic stimulation, there is constriction of the blood vessels leading to decreased intestinal perfusion [2]. Thus, even minimal changes in cardiac output (i.e., ejection fraction) can lead to significant intestinal ischemia [2]. Ischemia results in a decrease in intestinal motility and absorption and an increase in bowel wall permeability [3]. Bowel wall edema and decreased perfusion cause the gut wall to lose its integrity, becoming more permeable to bacteria, increasing rates of bacterial colonization. Food and nutrients are absorbed into enterocytes and later the capillaries via transcellular and paracellular transport. Sugar absorption studies show an increased absorption of transfer proteins in the small and large bowel, suggesting disruption of the intestinal membrane [2].

There is a phenomenon that occurs in end-stage CHF patients in which there is impaired absorption of fats and proteins resulting in severe unintentional weight loss. Cardiac cachexia is found in 16–42% of heart failure patients [2]. This finding is most pronounced in patients with functional class III and IV heart failure. These patients were measured to have an increased amount of protein and fat in their feces in comparison to patients without CHF [2]. Cardiac muscle wasting causes further decline of cardiac function. Cardiac cachexia is a predictive factor of increased mortality in patients with heart failure [2].

In heart failure, there is overactivity of the sympathetic system and a blunted parasympathetic response [4]. Although the sympathetic activity can be regulated pharmacologically with beta blockers and renin-angiotensin-aldosterone inhibitors, this is not the case for the parasympathetic system [4]. The parasympathetic vagus nerve innervates multiple organs including the heart and the GI tract [4]. Trials and studies have analyzed the use of vagal nerve stimulation (VNS) in advanced heart failure. Results show improvements in NYHA class, quality of life and end-systolic volumes measured via ECHO [4]. VNS has also been studied in patients with drug refractory gastroparesis, a chronic impairment in gastric motility and delayed emptying. Noninvasive cervical vagus nerve stimulation (nVNS) similar to implanted gastric electric stimulation (GES) shows reduction in symptoms of nausea, vomiting, early satiety, and abdominal bloating [5].

Cardiogenic cirrhosis, also known as congestive hepatopathy, occurs as a result of right-sided heart failure or in the case of our patient, biventricular heart failure [6]. The backup of blood from the right ventricle into the liver results in hepatic congestion and subsequent damage. Interestingly, the changes on the GI tract that occur with advanced heart failure are very similar to the ones that occur as a result of liver cirrhosis. These include delayed gastric emptying, slowed gut motility, increased bowel permeability, and bacterial translocation [6].

CMR revealed that our patient had LVNC, a structural abnormality in which the left ventricle has a thinner than normal compacted layer and trabeculae [7]. This defect occurs during embryonic development with the premature cessation of endomyocardial compaction within the left ventricle, resulting in the formation of trabeculations and deep intratrabecular recesses [8]. This can lead to cardiomyopathy; however, it is a rare incidental finding in healthy individuals with preserved LV function, which accounts for approximately 35% of LVNC cases [8]. In adults, the prevalence of LVNC is about 0.05% [8]. 3-4% of heart failure patients sent for ECHO are found to have LVNC [8]. LVNC is more commonly found in African Americans and in males, with average age of diagnosis being 40–50 years old [8]. Overall, about 8 forms of LVNC exist with varying heterogeneity [8]. The diagnosis is made with ECHO or CMR by measuring the ratio of noncompacted to compacted layers of the LV muscle wall, with a ratio of ≥2 : 1 meeting the criteria for LVNC [8]. Although the etiology of LVNC is uncertain, it is thought to be genetic in origin [8]. Research has shown links between abnormalities in certain genes, metabolic, mitochondrial, and chromosomal diseases, with the occurrence of LVNC [7]. The genetic defects most commonly associated with LVNC are of the sarcomere genes which are also involved with both dilated and hypertrophic cardiomyopathies [8].

Mitochondrial disorders also known as mitochondriopathies (MCPs) can be either syndromic or nonsyndromic and can present at any age, affect any organ, and have broad symptomatology [9]. The heart is one of the most commonly affected organs in MCPs [9]. Cardiac manifestations can include cardiomyopathies, LVNC, and arrhythmias [9]. Gastrointestinal manifestations of MCPs include dysphagia, gastroparesis, vomiting, malabsorption, diarrhea, pseudoobstruction, pancreatitis, and hepatopathy [10]. Over 50% of MCPs show some GI involvement although this is often underdiagnosed [10]. Episodic vomiting is a common manifestation and often first presenting symptom of mitochondrial encephalomyopathy, lactic acidosis, and stroke-like episode syndrome (MELAS) [10]. Gastroparesis is a less frequent manifestation of nonsyndromic MCPs and occurs due to a metabolic defect in the smooth muscle cells or from decreased parasympathetic innervation [10]. Hepatopathy in MCPs although more commonly seen in children may result in elevated liver enzymes and hepatic steatosis [10]. Involvement of the liver can be seen in up to 20% of patients with mitochondrial disorders [10].

## 4. Conclusion

Advanced stage heart failure is not a typical or well-studied cause of gastroparesis. Although gastric emptying studies demonstrated that our patient did indeed have gastroparesis, it is possible that he had components of both bowel wall edema and vagal nerve dysfunction from his enlarged heart, leading to delayed gastric emptying, abdominal discomfort, and formation of a gastric bezoar. The ultimate culprit was his failing heart. Better control of the patient's heart failure with recovery of function and improvement in ejection fraction may give some symptom relief. This can only be monitored by close patient follow-up. It will also be interesting to conduct more research in the future to investigate mitochondriopathies to understand the link between LVNC and gastroparesis.
